# A Few Thoughts on the Importance of Prophylactic Treatment in the Decay of Human Teeth

**Published:** 1861-03

**Authors:** Abr. Robertson

**Affiliations:** Wheeling, Va.


### *A few Thoughts on the Importance of Prophylactic Treatment in the Prevention of the Decay of Human Teeth.*

By ABR. ROBERTSON, D.D.S., MD., Wheeling, Va.

Perfect teeth are so important to life, health, comfort, and beauty of the human organization, that it cannot escape the observation of any one, whether professional man or layman. So fully is this fact recognized, that within a few years past, a host of several thousand men in this country alone, have risen up and professedly devoted themselves to the care, preservation, restoration, and artificial supply of these important organs. And they sustain three colleges and two or three times that number of journals devoted to the instruction of men, and of disseminating knowledge in this art, which they claim to be a branch of the "healing art."

Hitherto dentists have almost exclusively devoted their efforts and attention to arresting the decay in teeth, where it has already commenced, by filling and other operations, and of supplying the places of those already lost by artificial substitutes. But those who have observed most, and who have considered and studied the subject most carefully, are fast becoming convinced that such treatment is not sufficient; that operations on the teeth alone cannot fulfil the desired object. Operations cannot preserve the teeth in their original integrity, beauty and usefulness.

True, they can do much to preserve, and retain and restore to usefulness, teeth that have been partially, and even to a very great extent, destroyed by decay. Anything that has been repaired is not a perfect thing. Teeth that have been filled, and especially that have been filed, are always more or less defaced. Therefore, for the perfect object desired, preventive rather than reparative treatment is required.

But as dentists usually do not, and ought not, pretend to practice general medicine, and therefore have not the opportunity to apply the necessary prophylactic treatment; and as physicians do not, and from want of time and other circumstances generally cannot, and ought not to practice dentistry, a mutual obligation to the community, and to each other, rests on both physicians and dentists in this regard.

Physicians ought to be better prepared (and here I wish to disclaim all intention of invidiousness or reflection upon the medical profession; but I have heard some very eminent medical gentlemen say, with great complacency, "I know nothing about the diseases of the teeth,") to determine what teeth are capable of being preserved by dental operations; and they ought, too, when they observed the teeth of their patients wanting such operations, to recommend them to some good dentist, in whose integrity, judgment, and skill they have confidence. They should be prepared, too, to judge whether a dentist has the requisite qualifications to entitle him to such confidence. Promptness in giving, and urging such advice, if need be, is also important, for the success of such operations depends much on their being performed in time. And it is especially important that physicians, and more particularly country physicians, who are obliged to extract teeth, should be so qualified as to know when a tooth ought to be extracted and when not; for, for want of such knowledge, many useful and perfectly sound teeth have been extracted, sacrificed, because the patient wishes it done, under the false impression that the tooth offended, when the pain, or at least the cause of the pain, was in some other tooth or part.

Dentists should also have much more knowledge of the general principles of medicine and surgery than many—most of them—possess; that they may know what cases require general as well as special treatment; and when general treatment is necessary, recommend their patients to the care of their family physician, or other proper medical advisers.

*Preservation* is the province and duty of the physician; *restoration* that of the dentist. But how shall physicians prevent the decay and subsequent loss of the teeth of their patients? As already intimated, by prophylactics. When and how shall these be applied? Doubtless it may be done in many ways and at many times, but my present purpose is simply to call attention to one or two particulars, which seem to me to be pre-eminently important and plain, and which are of almost universal application. And here I will premise by saying that if the physician is called to a patient suffering from any disease, and has so treated him as to save his life, to restore his general health, to relieve him of his present suffering, he may have—he has—done a great good; but if he have, by any neglect or oversight, left him in such a state as in after life to be annoyed by pains and sufferings that might have been avoided, he has not done all the good he might have done, or that it was his duty to do.

It is almost a universal impression among mankind (at least in this country) that medicines injure the teeth—that they cause their decay.

Dentists almost daily hear remarks like this, and from all classes of people: "My teeth were perfectly sound until I was sick, and then I took so much medicine that it ruined them." A belief so universal as this never originates without some foundation, but still it is by no means certain that the conclusions are either correct or philosophical, and in the matter now under consideration, they are almost always wrong. Medicines but rarely injure the teeth. But few medicines, under any circumstances, can injure them. But that the teeth often decay more during and after sickness than before; that they often commence to show signs of decay, and even to give their possessors trouble immediately after a spell of sickness, is most unquestionably true, and and for the plainest reason.

The secretions of the mouth in their normal state are alkaline, the buccal mucus being slightly acid, while the secretions of the glands of the mouth—the saliva proper—which is by far the most abundant secretion, is alkaline. Decay of the teeth is caused by agents acting upon them chemically.

Most, if not all diseases, more or less change the character of all the secretions. The secretions of the mouth are changed from an alkaline to an acid reaction by many diseases, as gout, rheumatism, fevers, cachexias, &c., and especially by inflammation of the stomach and alimentary canal and indigestion. This effect is sometimes produced by suppressing the glandular secretions, and thus letting the acid mucus predominate; but more commonly by changing these secretions from alkaline to acid. While this altered state of the secretions continues, the teeth are constantly exposed to a bath, weaker or stronger, of acid, the only agent that is capable of injuring them, that is ever likely to come in contact with them while in the mouth. This acts upon them chemically and constantly; and this I am satisfied—not only from the general belief that medicines injure the teeth, (though from what has already been said it is clear that proper medicines properly administered, that is, so administered as to restore these altered secretions, protect instead of injuring these organs,) induced by the observation that the teeth so generally fall into decay after the occasion for taking medicines—after "a spell of sickness"—but from many years'

experience and observation in the cure and treatment of the teeth—is *the great* cause of their decay. True the continued use of acids, and especially of the mineral acids given as medicines, and also the topical use of such salts as have less affinity for their acid bases than has the lime of the teeth, sometimes effect sad ravages in these organs.

From the foregoing remarks it seems to me plain what course should be pursued; what is the only course to preserve the teeth of the community in their original integrity; or, at least, the course that will do much, very much, towards securing that most to be desired result.

First, that whenever a patient comes under the treatment or care of a physician, he should not only seek to restore health by restoring the secretions to their normal condition; but that he should carefully observe and satisfy himself by suitable tests, of which litmus paper is perhaps the most convenient, whether the secretions of the mouth are acid; and if so, take care to protect the teeth and other parts from its injurious effects while that condition lasts, by prescribing the frequent use of such antacid, or stimulating gargles, or washes, as shall most effectually counteract or prevent its action on the teeth, as well as to direct that they be kept as free as possible from all accumulations as might, by neglect, ferment and become acid in the mouth.

And second, when acids or salts whose acids have stronger affinities for lime than for their own constituents, are exhibited, by directing that they be taken through tubes so as to prevent their contact with the teeth, or by immediately following their exhibition by some antacid wash, as solution of the carb. sodæ. Both might be but a prudent precaution.

If this course were generally adopted by physicians, and more especially if the community generally were instructed in the importance of such precautions, I am well assured that we should cease to hear such sad complaints of the bad effects of medicines on the teeth; and that a vast amount of expense, discomfort, pain and deformity would be avoided.—*Medical Monthly.*

